# Development and validation of a four-dimensional registration technique for DCE breast MRI

**DOI:** 10.1186/s13244-022-01362-w

**Published:** 2023-01-26

**Authors:** Chiara Mattusch, Ulrich Bick, Florian Michallek

**Affiliations:** 1 Charité – Universitätsmedizin Berlin, corporate member of Freie Universität Berlin and Humboldt-Universität zu Berlin, Department of Radiology, Charitéplatz 1, 10117 Berlin, Germany; 2 Department of Radiology, Mie University Graduate School of Medicine, Tsu, Japan

**Keywords:** MRI, Dynamic contrast-enhanced, Principal component analysis, Registration, Breast cancer

## Abstract

**Background:**

Patient motion can degrade image quality of dynamic contrast-enhanced magnetic resonance imaging (DCE-MRI) due to subtraction artifacts. By objectively and subjectively assessing the impact of principal component analysis (PCA)-based registration on pretreatment DCE-MRIs of breast cancer patients, we aim to validate four-dimensional registration for DCE breast MRI.

**Results:**

After applying a four-dimensional, PCA-based registration algorithm to 154 pretreatment DCE-MRIs of histopathologically well-described breast cancer patients, we quantitatively determined image quality in unregistered and registered images. For subjective assessment, we ranked motion severity in a clinical reading setting according to four motion categories (0: no motion, 1: mild motion, 2: moderate motion, 3: severe motion with nondiagnostic image quality). The median of images with either moderate or severe motion (median category 2, IQR 0) was reassigned to motion category 1 (IQR 0) after registration. Motion category and motion reduction by registration were correlated (Spearman’s rho: 0.83, *p* < 0.001). For objective assessment, we performed perfusion model fitting using the extended Tofts model and calculated its volume transfer coefficient *K*^trans^ as surrogate parameter for motion artifacts. Mean *K*^trans^ decreased from 0.103 (± 0.077) before registration to 0.097 (± 0.070) after registration (*p* < 0.001). Uncertainty in perfusion quantification was reduced by 7.4% after registration (± 15.5, *p* < 0.001).

**Conclusions:**

Four-dimensional, PCA-based image registration improves image quality of breast DCE-MRI by correcting for motion artifacts in subtraction images and reduces uncertainty in quantitative perfusion modeling. The improvement is most pronounced when moderate-to-severe motion artifacts are present.

**Supplementary Information:**

The online version contains supplementary material available at 10.1186/s13244-022-01362-w.

## Key points

- PCA-based registration improved motion-related image quality according to subjective and objective criteria.
- The impact of registration was positively correlated with motion severity.
- Registration improved perfusion quantification by reducing model-related uncertainty.

**Supplementary Information:**

The online version contains supplementary material available at 10.1186/s13244-022-01362-w.

## Background

Breast cancer is the leading cancer in women and, after lung cancer, the second most frequent cause of cancer death in women [1]. Screening methods targeting the general population and first-line diagnostics are clinical examination, sonography, and especially mammography [2]. Moreover, magnetic resonance imaging (MRI) using dynamic contrast-enhanced (DCE) sequences has shown excellent precision in detecting and characterizing cancer lesions in the breast [3]. It has therefore been recommended that patients with a high lifetime risk of developing breast cancer undergo an annual screening with DCE-MRI [4]. To overcome MRI-inherent challenges, including high-cost infrastructure and long examination time, shortened protocols with or without diffusion-weighted imaging (DWI) have been used [5, 6]. Nevertheless, due to its excellent sensitivity, DCE-MRI is a highly desirable imaging modality in the clinical setting [7].

Motion artifacts are a pertinent challenge in clinical routine protocols, jeopardizing image quality when interpretation relies on subtracted images. To address this issue, image registration, a constituent of image preprocessing [8], can be performed to compensate for patient motion [9]. In the literature, several registration methods have been proposed [10]; however, most algorithms use a three-dimensional time-point-by-time-point approach, neglecting variations in image intensity introduced by contrast agent kinetics [11]. To incorporate perfusion as the physiological determinant of temporally variable contrast agent kinetics, the authors of a previous technical study in a limited number of patients have proposed a principal component analysis (PCA)-based registration scheme for application to breast MRI [12]. This approach operates simultaneously on all four dimensions (three spatial dimensions and one temporal dimension) and integrates signal variation that occurs in anatomical structures over time due to perfusion characteristics [13].

Our study aimed at validating a PCA-based registration approach in four-dimensional DCE breast MRI to account for contrast agent kinetics in a clinically well-characterized population of women with breast cancer. We evaluated the impact of image registration on clinical lesion conspicuity and perfusion analysis using both qualitative and quantitative parameters.

## Methods

### Patient selection

**Fig. 1:**
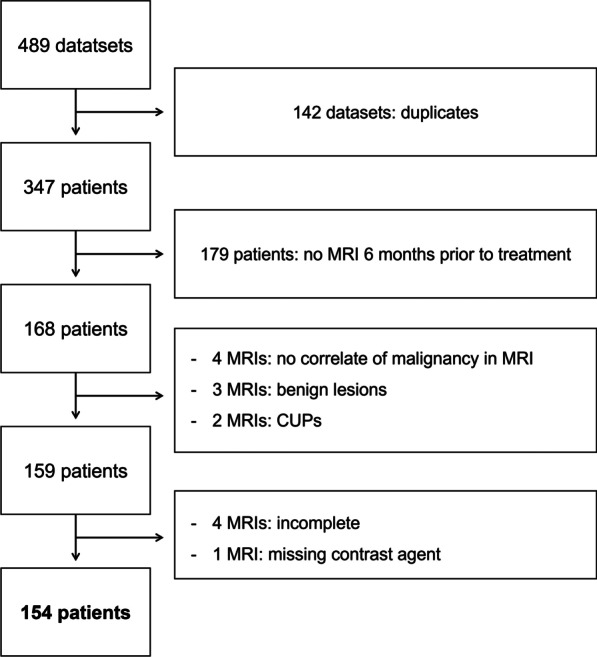
Selection of study population. Initially, we identified datasets of patients with breast cancer who underwent initial or follow-up DCE-MRI at our department in 2018 and 2019. After exclusion of duplicate datasets, necessary when patients underwent multiple breast MRI examinations during the study period, further criteria had to be met, including a pretreatment DCE-MRI within 6 months of initial diagnosis. *CUP* Cancer of unknown primary

The study was approved by the local institutional review board and written informed consent of patients was waived. We derived our retrospective patient cohort from a total of 489 breast cancer patients with primary diagnosis at our site who were referred for initial or follow-up MRI in 2018 and 2019. The indications for breast MRI at our institution were high-risk screening in patients with pathogenic variants in known breast cancer risk genes and patients with calculated high-risk profile [14], surveillance MRI in patients with a history of prior breast cancer, and tumor staging in patients with a newly diagnosed breast cancer. After removing duplicates from our dataset, e.g., due to more than one follow-up MRI, we excluded patients who did not undergo DCE-MRI before treatment or whose most recent pretreatment MRI was not within a six-month range of initial diagnosis. Additionally, patients were excluded if pretreatment MRI did not show malignant lesions, e.g., due to cancer of unknown primary with a histopathological breast cancer diagnosis, or if benign lesions were diagnosed after initially suspected malignancy, or if patients exclusively had non-mass lesions. Furthermore, we excluded one patient because MRI was performed without contrast agent and four patients because imaging datasets were incomplete. The final study population included 154 patients with complete imaging and histopathological characterization. Figure 1 depicts the selection of the study population in a flowchart.

### Imaging

Imaging was performed on 1.5 Tesla scanners and included unenhanced T1-weighted and T2-weighted sequences, a DCE sequence using a T1-weighted gradient echo sequence at 6 time points (unenhanced, 90 s after contrast agent administration and four subsequent timepoints every 70 s), and a fat-saturated delayed enhancement sequence. DCE sequences, which were subjected to image registration, were performed using a standard gradient echo sequence in primary three-dimensional acquisition with typical imaging parameters as follows: image matrix 512 × 512px with 0.7 mm in plane pixel spacing, slice thickness 2 mm, echo time 4.7 s, repetition time 7.5 s, and flip angle 25°.

### Registration

Similar to a registration protocol previously reported in the literature [12, 13], we implemented a PCA-based registration protocol operating on four-dimensional DCE-MRI input images. We used the open-source SimpleElastix framework and its Python 3.6 links [15]. Our registration protocol employed a non-rigid B-spline transformation in a multi-resolution approach incorporating four individual resolutions with a scaling pyramid schedule of eightfold, fourfold, twofold down-sampling and, finally, the original resolution to account for motion on different spatial scales (i.e., addressing coarse motion first before correcting fine motion). We employed a registration metric based on principal component analysis as previously described and validated for registering dynamic contrast-enhanced imaging datasets (PCA2) [16]. This registration method attempts to separate image intensity variations due to physiology (i.e., contrast agent kinetics) from anatomical motion by compacting the eigenvalues of the correlation matrix between temporally resolved image volumes. The correlation matrix is assumed to be dispersed in the presence of motion, thereby overweighting less dominant eigenvalues. By reducing less dominant eigenvalues, the contribution of motion is alleviated, leading to an improvement of spatial alignment with retained intensity variation over time. Since the method operates in a groupwise manner, it does not rely on manually selecting a reference image. Registration was performed at a standard clinical workstation. Afterward, we calculated subtraction images for each time point after contrast administration by subtracting the unenhanced image from the enhanced images. Both unregistered and registered images were subjected to further processing as outlined below.

### Segmentation

Tumor segmentation was performed as a prerequisite for quantitative assessment of motion. Regions of interest (ROIs) were selected semiautomatically; in the subtracted images, we identified the time point with earliest lesion enhancement and optimal lesion conspicuity, which, in most cases, was the first postcontrast time point. Only when contrast enhancement was markedly delayed, the second postcontrast time point was chosen. For semiautomatic segmentation, we performed intensity thresholding with an intensity threshold of 0.1 of maximum lesion intensity after subtraction. For voxel selection, we used a spheroid brush tool with an arbitrarily adjustable diameter according to individual lesion size. Voxels with intensity values above the threshold were included in the ROI. Further ROI adjustment was done using three-dimensional morphological dilation or erosion, as deemed necessary. All segmentations were performed in a consensus procedure of two readers (a qualified physician and a board-certified radiologist with six years of experience in breast MRI). In case of disagreement or in non-trivial cases, a senior reader was consulted (> 30 years of experience).

### Qualitative assessment of motion

Motion in the DCE-MRI sequences was quantified for registered and unregistered images in a two-reader consensus process (C.M. and F.M.). Readers were blinded to whether image processing had been performed. Additionally, 30 randomly selected cases and cases of disagreement were discussed with a senior consultant (U.B.). A numerical ranking scale (NRS) from 0 (no motion) to 10 (nondiagnostic due to severe motion) was defined. Based on the fine-granular NRS, four clinically more manageable motion categories were defined to grade diagnostic quality and to represent the level of clinical impairment introduced through varying amounts of motion artifacts (Table 1). Motion assessment was performed on a per-patient level.

**Table 1:** Description of numerical ranking scale and motion categories

NRS	Motion category		Motion pattern description
0	0	No motion	No motion
1	1	Mild motion	Delicate budging
2			Discreet, yet progressive jolt or small budging
3			Relevant, progressive budging
4	2	Moderate motion	Shift between *t*0 and *t*1, relevant subtraction artifacts
5			Moderate unilateral motion
6			Significant unilateral shift and/or rotational component
7			Large *t*0–*t*1 shift, substantial subtraction artifacts
8	3	Severe motion (nondiagnostic image quality)	Rugged distortions, tumors possibly not detectable
9			Extensive lateral/rotational motion with artifacts
10			Maximum and extensive distortions

Intrasequence motion was assessed on a numerical ranking scale and assigned to one of four motion categories. Use of this ranking scale ensures quantifiability and reproducibility of subjective assessment of motion, both before and after registration. Steps on the NRS are described by typical examples of respective motion patterns. *t*0–*t*1 shift refers to disproportionately strong motion between the unenhanced time point [*t*0] and the first postcontrast time point [*t*1]

*NRS* Numerical ranking scale

### Quantitative assessment of motion

Quantitative assessment of motion was performed in the previously segmented ROIs. We quantified the impact of registration by calculating residual errors of perfusion modeling using the extended Tofts perfusion model as previously described [13, 17–19]. This model-based similarity measure accounts for the physiological relevance of signal variation through perfusion and, therefore, is a meaningful measure for assessing registration quality [19]. Low levels of motion are expected to yield small residual errors. Therefore, reducing motion through registration is expected to decrease residual errors. We calculated residual errors for tumors on a voxel-by-voxel basis, which enabled percentile-wise assessment. The change in residual values was calculated as the difference of postregistration minus preregistration with negative values indicating motion reduction. Moreover, we calculated volume transfer constant *K*^trans^ using the extended Tofts model. Perfusion modeling was performed using software, which has been developed in-house and has been applied in previous research [20–22].

### Statistical analysis

Demographic data, observer NRS values, and residual errors were analyzed by descriptive statistics. The data were not normally distributed, therefore we tested for significance using the sign test and the Wilcoxon signed-rank test. Correlation of paired samples was assessed with Spearman’s rank test. Subgroup analyses were performed by tumor entity and by tumor T-stage, each with a nonparametric ANOVA test. Tumor entities are listed in Table 2. For subgroup analysis by T-stage, tumors were categorized according to TNM criteria, i.e., DCIS, tumor < 2 cm, tumor 2–5 cm, and tumor > 5 cm. A level of *p* ≤ 0.05 was considered statistically significant. Statistical analysis was performed with IBM SPSS Statistics for Windows (Version 27.0, released 2020. IBM Corp. Armonk, NY).

**Table 2:** Demographic and histopathological characterization of the study population

Characteristic	Value
Number of included lesions	160 in 154 patients
Patient age, mean (range) in years	47 (24–75)
High-risk patients*, *n* (%)	80 (50)
Tumor size, median (range) in mm	15.5 (0.9–170)
Lymph node involvement, *n* (%)	48 (30)
Focality/centricity, *n* (%)	
Unifocal	127 (79)
Multifocal	19 (12)
Multicentric	14 (9)
Tumor entity, *n* (%)	
Ductal carcinoma in situ (DCIS)	14 (9)
Invasive carcinoma of no special type (NST)	110 (69)
Invasive lobular carcinoma	27 (17)
Invasive papillary carcinoma	2 (1)
Mucinous carcinoma	2 (1)
Tubular carcinoma	2 (1)
Medullary carcinoma	2 (1)
Carcinoma with medullary features	1 (1)
Precancerous component, *n* (%)	
DCIS component	96 (60)
LCIS component	6 (4)
None	58 (36)
Grading, *n* (%)	
G1	24 (15)
G2	90 (56)
G3	46 (29)
Immunohistochemical subtype, *n* (%)	
Luminal A	34 (21)
Luminal B	85 (53)
Triple-negative	22 (14)
HER2-positive	2 (1)
Unknown	3 (2)
Not applicable (DCIS)	14 (9)

Rounding was performed to the nearest percent. Values are calculated on the per-lesion level. Information on proliferation index and hormone receptor expression is provided in the additional file (Additional file 1: Tables S1 and S2)

*LCIS* Lobular carcinoma in situ

*BRCA1/2 carriers and high-risk BRCA1/2 noncarriers are considered high-risk patients according to Bick et al. [14]

## Results

### Patient population

Our patient population consisted of 154 women with a total of 160 breast lesions. Five patients had bilateral tumors, and one patient had two histologically different unilateral tumors. Patient age at diagnosis ranged from 24 to 75 with a median of 47 (interquartile range, IQR 15). Median tumor size determined clinically or histologically was 15.5 mm, ranging from 0.9 to 170 mm (IQR 19.8). Radiologically determined, mean tumor volume was 5.4 ml (standard deviation, SD 9.6). The most common tumor entities were carcinomas of no special type (NST) and invasive lobular carcinomas, which accounted for 69% and 17% of tumor entities, respectively. Being either BRCA1/2 carriers or high-risk BRCA1/2 noncarriers, 50% of our study population were high-risk patients and were included in the intensified surveillance program at our center [14]. Further characteristics of our histologically well-defined patient population are given in Table 2.

### Subjective assessment of motion reduction

**Fig. 2:**
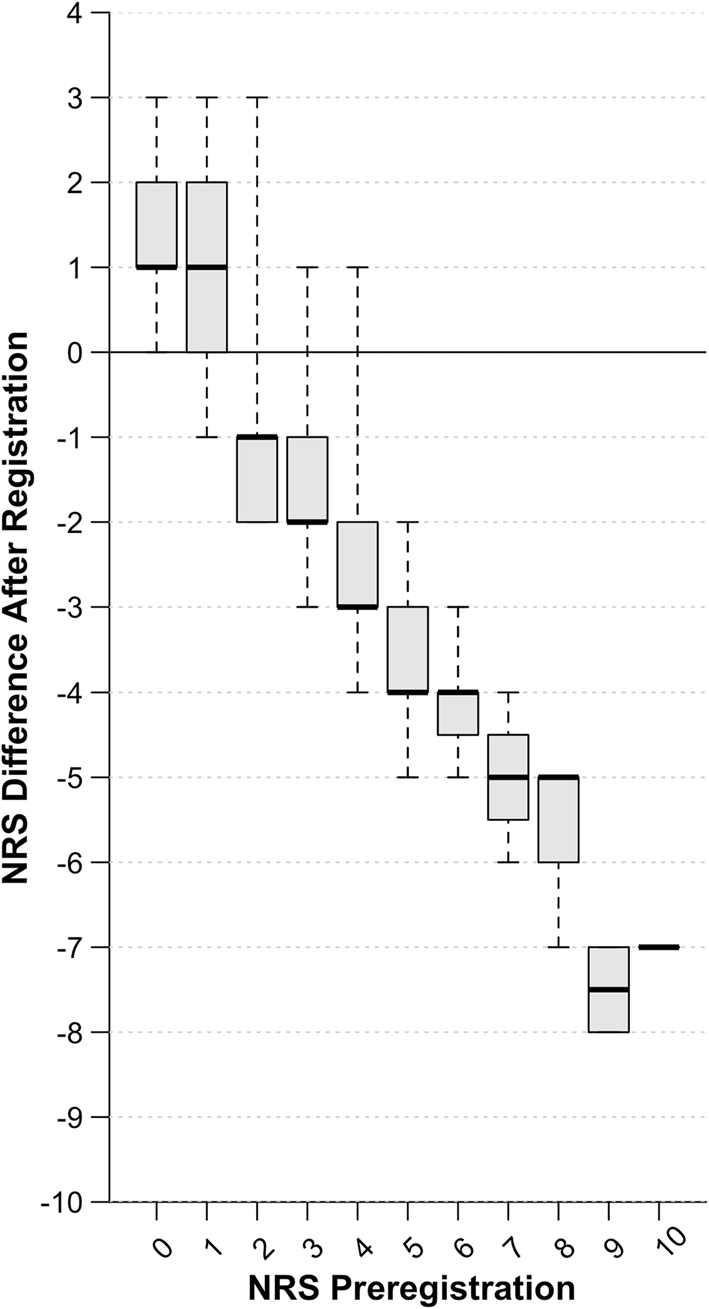
Subjective assessment of impact of registration. Observers assessed motion using a numerical ranking scale, classifying images according to their extent of motion before and after registration. The attained impact on the NRS by registration is shown in correlation to the initially rated extent of motion. *NRS* Numerical ranking scale

**Fig. 3:**
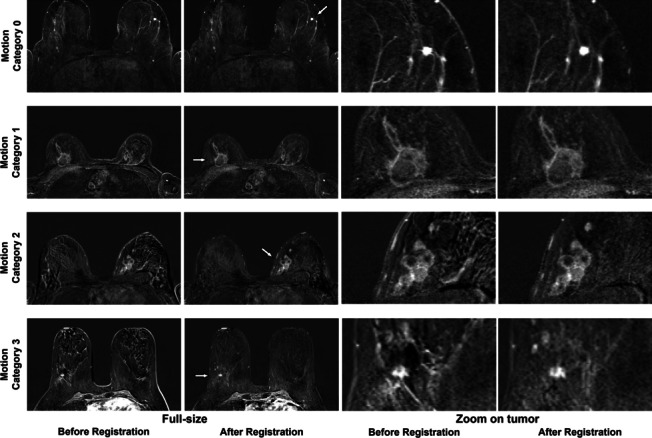
Illustration of motion categories. Readers subjectively assigned images to one of four motion categories based on visual assessment. Each motion category is illustrated by an example shown at the fifth time point after contrast agent injection. The higher the initial motion category, the greater was the visually perceived impact of motion on image quality and hence, potential diagnostic limitation. The unenhanced image was subtracted from the enhanced images. Lesions are indicated by an arrow in the second column (full-size images after registration). The respective areas were enlarged and are shown in columns three and four, before and after registration, respectively

**Fig. 4:**
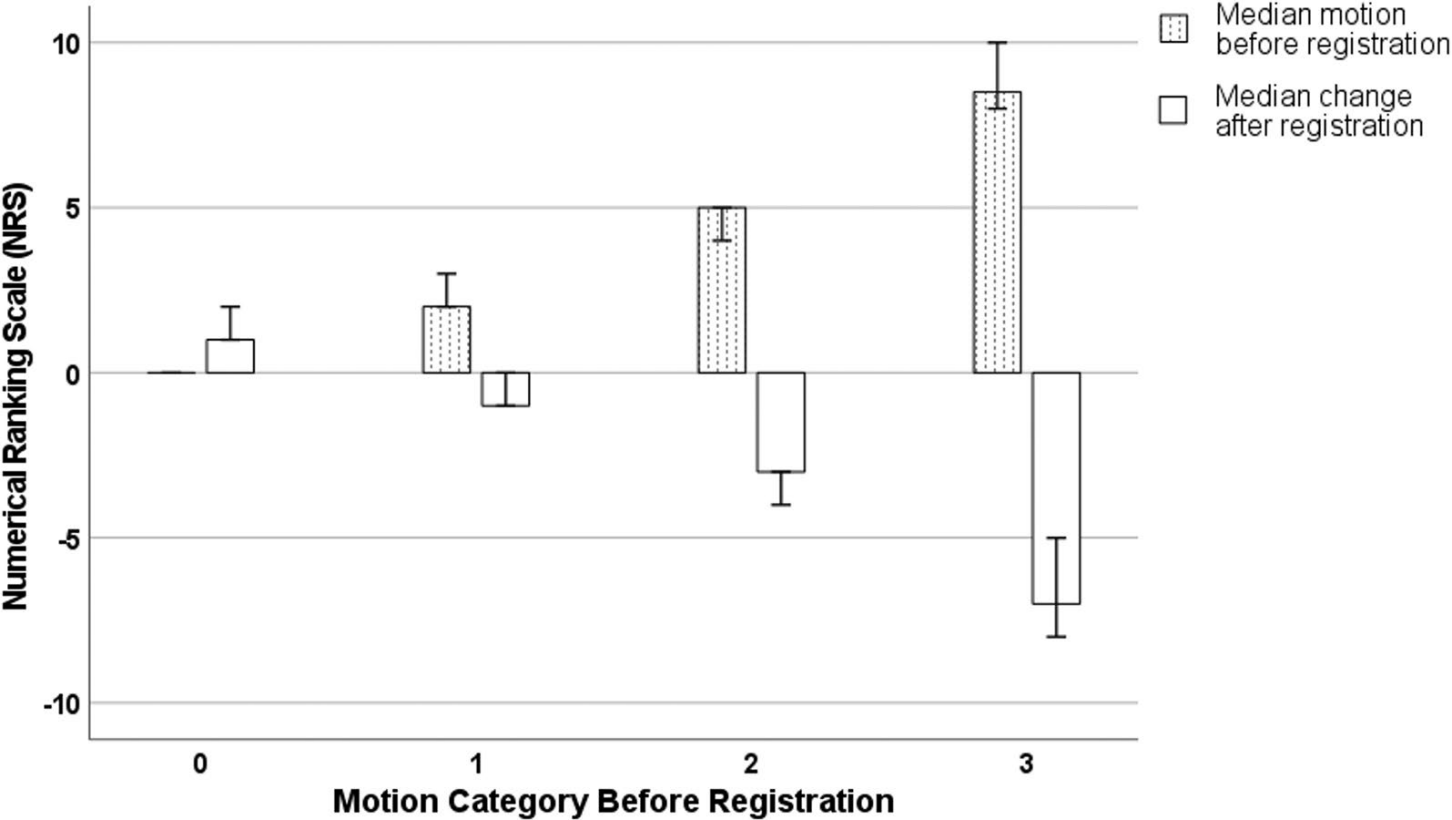
Impact on registration by motion category. Cases are grouped by motion category before registration. Dotted bars represent median motion on the NRS before registration. Blank bars indicate median change in motion after registration. A 95% confidence level is indicated. Registration resulted in a nonsignificant NRS change for cases initially assigned to categories 0 and 1 (*p* = 0.65). In DCE-MRI datasets of patients with initially impaired diagnostic quality due to motion (categories 2 and 3), a significant decrease in the NRS was demonstrated for each category after registration (median NRS change for motion category 2: − 3, IQR 1, *p* < 0.001; median NRS change for motion category 3: − 7, IQR 2, *p* < 0.001)

The duration of registration was relatively constant, and the median time required was 11 min and 15.3 s (IQR 25.0 s). Before registration, the median NRS was 3 with an IQR of 3. After registration, the median NRS decreased to 1 with an IQR of 1 (*p* < 0.001). The extent of motion reduction on the NRS depended on preregistration NRS values, which is visualized in Fig. 2. There were 17 patients (11.0%) in motion category 0, 70 patients (45.5%) in motion category 1, 61 patients (39.6%) in motion category 2, and six patients (3.9%) in motion category 3. Examples illustrating the four motion categories are shown in Fig. 3. In MRIs of patients initially categorized as none or limited motion (motion categories 0 and 1), registration resulted in a combined median change of 0 (IQR 3), i.e., no visual change was observed. However, in the subgroup combining patient cases of motion categories 2 and 3, showing moderate or severe motion artifacts, a significant median decrease in 3 (IQR 1, *p* < 0.001) on the NRS was found after registration. Sixty-six of the 67 cases in the latter subgroup were assigned to categories 0 and 1 after registration, and the median motion category decreased from 2 (IQR 0) to 1 (IQR 0). The impact of the motion category assigned prior to registration on the result of registration was significant (Spearman’s rho 0.83, *p* < 0.001), as displayed in Fig. 4.

### Objective assessment of motion reduction

**Fig. 5:**
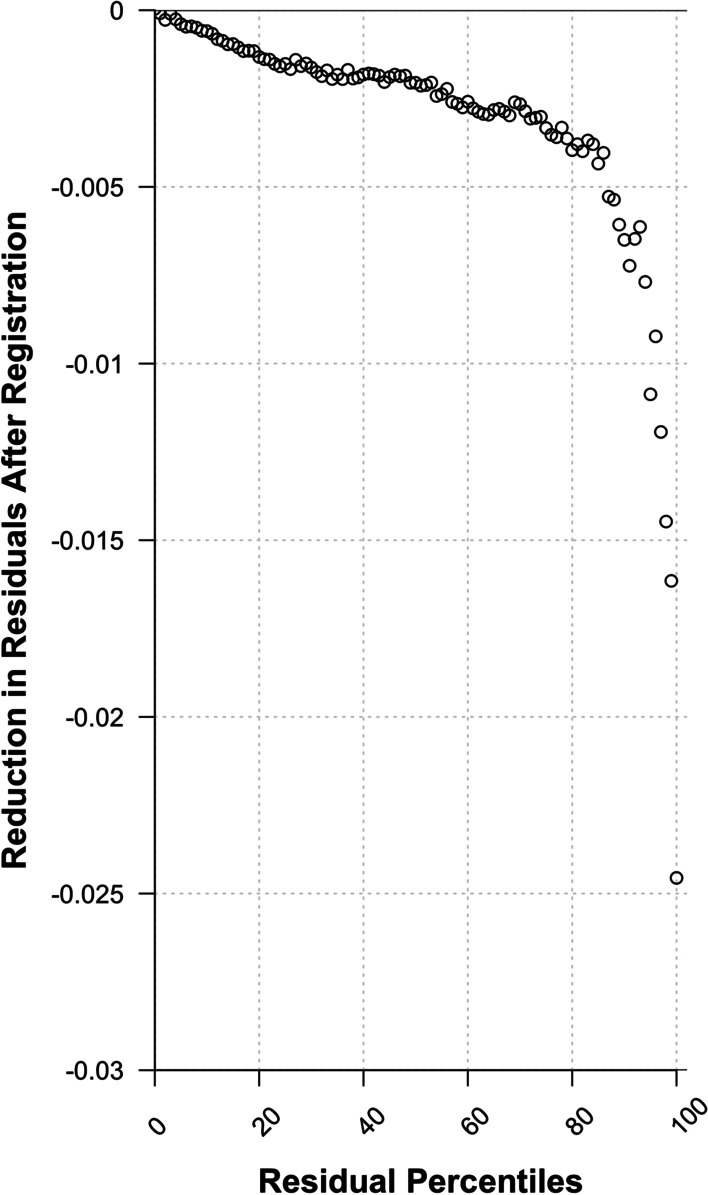
Impact of registration on residuals by intralesion percentiles. Difference in voxel-wise residuals from perfusion model fitting using the extended Tofts model. Registration resulted in a reduction in residuals, which was especially pronounced in areas with high residual errors prior to registration (around the 80th percentile and higher)

**Fig. 6:**
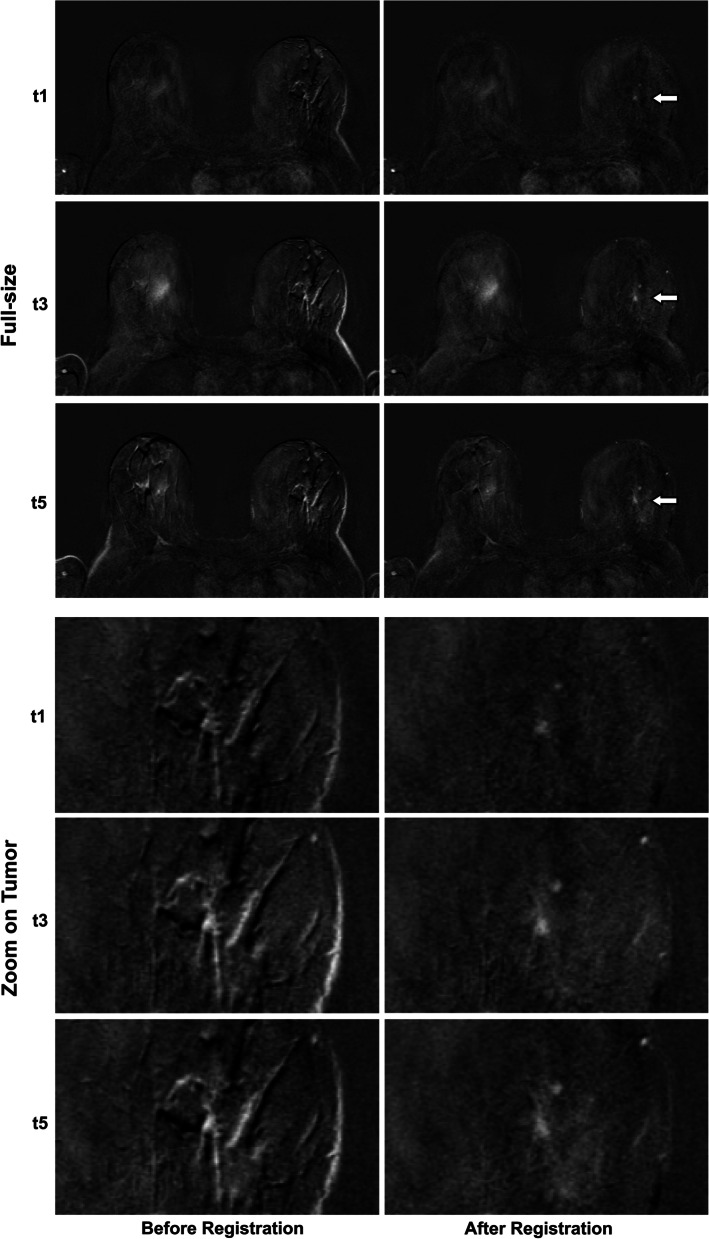
Case history of a 48-year-old patient with ductal carcinoma in situ (DCIS) of 18-mm diameter. Tumor histopathology showed a G1 tumor, 10% progesterone receptor expression, and 100% estrogen receptor expression. The patient was not considered a high-risk patient. Images show subtractions of the same axial plane at three different time points (the first [*t*1], third [*t*3] and fifth [*t*5] time point after contrast administration), first in full-size and beneath by zooming in on the tumor. The left column shows images before registration and the right column after registration. In subjective assessment, the case was assigned to motion category 3 with an NRS (numerical ranking scale) value of 9 (defined as “nondiagnostic image quality”). After registration, the previously undetectable tumor in the subtracted images is unveiled (indicated by arrows in full-size images). Following registration, the images were assigned to motion category 1 with a NRS of 1. Quantitative analysis showed for the 90th percentile of intralesional motion, a 0.021 reduction in residual errors. Median *K*^trans^ decreased from 0.0482 (IQR 0.0561) before registration to 0.0438 (IQR 0.0423) after registration

Registration resulted in a significant reduction in the mean volume transfer coefficient *K*^trans^ from 0.103 (SD 0.077) before registration to 0.097 (SD 0.070) after registration (*p* < 0.001). Standard deviation of *K*^trans^ decreased by 7.4% (SD 15.500, *p* < 0.001) from 0.096 (SD 0.064) before to 0.087 (SD 0.057) after registration. Analysis of residual errors revealed a moderate, but significant reduction of the 50th percentile of residuals (mean 0.0034, SD 0.0075), visualized in Fig. 5. For higher percentiles, which represent areas with high perfusion modeling uncertainties, a marked reduction in residuals was demonstrated, e.g., the 90th percentile of residual errors showed a mean reduction of 0.0273 (SD 0.1157). Before registration, residual errors of the 90th percentile ranged from 0.032 to 1.801 with a mean of 0.2398 (SD 0.2307), whereas after registration, the range was reduced to 0.027 and 1.226, respectively (mean 0.2125, SD 0.1745). Moreover, we found that on average, tumors with higher residual errors consistently showed higher levels of *K*^trans^ for all percentiles (Spearman’s *ρ* = 1.00, *p* < 0.001). For example, in locations with average residual errors (exemplarily represented by the 50th percentile of intralesion residuals), *K*^trans^ showed a moderate correlation with the extent of residual errors (Spearman’s *ρ* = 0.596, *p* < 0.001). A representative case is shown in Fig. 6.

### Subgroup analyses

No significant differences were found by tumor entity, for neither quantitative (*p* = 0.586) nor qualitative assessment (*p* = 0.456). For subgroup analysis by T-stage, qualitative assessment did not show significant differences (*p* = 0.897). However, a significant difference in quantitative assessment was found (*p* < 0.001): T-stage was positively correlated to the reduction of residuals (Spearman’s *ρ*: − 0.32, *p* < 0.001) showing higher benefit of registration in tumors with higher T-stage.

## Discussion

In our study, we investigated the effects of a previously established four-dimensional registration method that incorporates signal variations over time and accounts for perfusion characteristics on motion artifacts in breast DCE-MRI datasets. We found that motion artifacts were reliably reduced both in subjective qualitative assessment and objective quantitative measurement by perfusion modeling using the extended Tofts model [17]. Motion reduction was especially effective in areas with high perfusion modeling uncertainty, measured in terms of residual errors in the fitting process. Furthermore, our registration improved visual perception of motion artifacts and, thus, image quality. Results strongly correlated with perceived motion in the original, unregistered images, showing major improvement for images with initially strong motion artifacts and a minor impact for images with little or no motion artifacts.

Early registration techniques optimized for breast DCE-MRI were exclusively based on rigid and affine transformation, correcting for rotational and translational misalignment of images [23]. In contrast, non-rigid registration methods attempt to anticipate motion with free-form deformation based on B-splines [24], and various refinements of these methods have been published [12, 25–29]. Recently, research in breast imaging registration has focused on multimodality registration including cross-modality registration between mammography [30–33], FDG-positron emission tomography (PET) [34, 35], and ultrasound [36] with DCE-MRI. Meanwhile, intrasequence registration has been implemented as a basis for subsequent image processing, such as multimodality registration [37–39]. However, in clinical reading routines, the potential benefit of registration is often ignored, although it might lead to diagnostic image quality of imaging datasets that would otherwise have nondiagnostic image quality. Registration algorithms accounting for perfusion characteristics have been found to effectively reduce motion, thereby minimizing artifacts and improving image quality [13]. Nevertheless, intrasequence registration incorporating physiological contrast enhancement patterns have not yet been evaluated in large, clinically well-characterized patient populations [12, 13]. Our study provides evidence that said registration technique could offer excellent subjective results in diagnostic reading settings of DCE-MRI datasets acquired in women with breast cancer, especially when pronounced motion is present. Hence, images necessitating motion reduction most to either facilitate, improve, or even enable a clinical diagnosis benefit strongly from the implementation of this registration technique. Figure 4 illustrates the impact of registration in a case with severe motion artifacts, which completely masked the tumor in subtraction images before registration. Furthermore, when a patient’s clinical diagnosis requires higher density of imaging data, longer acquisition protocols can increase spatial or temporal resolution, in turn potentially leading to improved imaging quality or perfusion quantification stability, respectively. On the other hand, the implementation of longer protocols can result in a lower signal-to-noise ratio (SNR) and an increase in motion artifacts [40]. Other methods have been devised, such as super-resolution reconstruction (SSR), to densify imaging data without a need for longer scan duration [41]. However, in a clinical setting, the occasional need for higher spatial resolution could be met by using slightly longer acquisition protocols and consecutively offsetting a possible increase in motion artifacts by applying PCA-based registration.

The impact of registration on perfusion quantification—namely perfusion model parameters like *K*^trans^ and calculation of uncertainties using error estimates such as residual error—has been investigated in a recent study [18]. The results of our study show a significant decrease in voxel-wise residual error after registration when the extended Tofts perfusion model is applied. This effect was more pronounced for voxels with high residual error prior to registration, which correspond to areas with higher perfusion variation. We found a distinct reduction in residuals after registration especially at the 90th percentile of voxel-wise residuals, which is in accordance with previous studies [18]. The challenge in applying pharmacodynamic parameters to the evaluation of registration techniques has been previously identified by Tofts et al. and Mouawad et al., who have found *K*^trans^ to be rather insensitive to voxel misalignment and its reduction by registration [18, 42]. Nevertheless, our results show a significant reduction of mean *K*^trans^ and a significant reduction in uncertainty in voxel-wise perfusion quantification, underlining the efficacy of perfusion-guided registration techniques. It has been suggested that minor quantitative motion reduction has major benefits not only for visual assessment, but also for pharmacokinetic modeling [43]. Physiology-based, quantitative assessment parameters should be used to quantify the impact of registration, especially when the registration protocol is based on pharmacokinetics of contrast agent.

The deduction of histopathological characteristics by evaluating perfusion parameters in breast imaging has been investigated before. However, image registration methods which integrate perfusion characteristics, e.g., by using four-dimensional PCA-based registration protocols, were not frequently applied, instead respective studies often relied on affine registration algorithms [37, 44]. After all, image registration is a prerequisite for quantitative analysis of DCE images, using either machine learning or radiomics algorithms, and might be viewed as mandatory for valid model building [45]. Omitting the registration step or using suboptimal techniques during preprocessing might jeopardize quantitative results due to spatiotemporal incoherence of image information.

Our study has limitations, including its retrospective study design and a study population exclusively comprising patients with precancerous or malignant breast lesions. Due to availability of an established histology, we selected malignant lesions as our clinical target condition, as one of our objectives was to evaluate the impact of image registration on perfusion quantification in focal lesions. Although the composition of our study population does not allow us to draw conclusions on registration effects in patients with benign findings or normal scans, the results account for a broad, histologically well-defined, and clinically derived study population (with a high percentage of pathogenic variants), which in turn increases the transferability to patients with breast cancer or precancerous lesions. Additionally, the exclusion of benign lesions prevented the introduction of a potential selection bias as many typically benign lesions are not referred for biopsy at our institution. Furthermore, the benefit of the registration technique investigated here has been strictly validated against preregistration images. This corresponds to a typical clinical imaging analysis process, as registration is frequently not used routinely, disregarding its undisputable value. In this context, Melbourne et al. have recently shown in their preliminary study the superiority of a four-dimensional, perfusion-based registration technique over standardly applied three-dimensional, non-rigid registration techniques [12]. Although our work has yielded significant results, we presume that further optimization of the registration technique can be attained by identifying ideal parameter settings, such as Mehrabian et al. have demonstrated in a complex, automated process to establish a set of validated parameter settings for deformable registration [46]. This approach could further improve the scope of image alignment and should be investigated in future research, especially when considering the inclusion of DCE-MRI sequences with fat saturation. Finally, we did not yet implement our registration technique in a prospective setting to demonstrate its clinical value in a routine workflow.

## Conclusion

In this study, we have demonstrated clinical applicability of a perfusion-based, four-dimensional registration method of breast DCE-MRI in a large histopathologically well-characterized patient population with breast cancer and precancerous breast lesions. The impact of registration on image quality has been shown both quantitatively and qualitatively. Our results suggest that image registration might improve clinical reading and reduce uncertainty in quantitative parameter assessment, e.g., by perfusion modeling.

## Supplementary Information

**Additional file 1. Table S1.** Proliferation index. **Table S2.** Immunohistochemical receptor constellation.

## Data Availability

The datasets used and analyzed during the current study are available from the corresponding author on reasonable request.

## Abbreviations

CUP: Cancer of unknown primary
DCE: Dynamic contrast-enhanced
DCIS: Ductal carcinoma in situ
DWI: Diffusion-weighted imaging
IQR: Interquartile range
LCIS: Lobular carcinoma in situ
MRI: Magnetic resonance imaging
NRS: Numerical ranking scale
NST: Carcinoma of no special type
PCA: Principal component analysis
PET: Positron emission tomography
ROI: Region of interest
SD: Standard deviation
