# Supplementary material for: Development and validation of a four-dimensional registration technique for DCE breast MRI
Source: Insights Imaging. 2023 Jan 26;14:17. doi: 10.1186/s13244-022-01362-w (PMC9880129; doi:10.1186/s13244-022-01362-w)
Supplement: Supplementary file 1 — Additional file 1. Table S1. Proliferation index. Table S2. Immunohistochemical receptor constellation. [file 13244_2022_1362_MOESM1_ESM.pdf]

ELECTRONIC SUPPLEMENTARY MATERIAL

Development and validation of a four-dimensional registration technique for DCE breast MRI

Supplemental Table 1. Proliferation index.

Proliferation Index	n (%)
**Negative, n (%)**	26 (16)
**High, n (%)**	3 (2)
**Very high, n (%)**	131 (82)

Proliferation was considered negative at a level lower than 10%, high when ranging from 10 to 25% and very high when exceeding 25%. Data of 4 (3%) lesions is unknown. Precancerous lesions were not included in the analysis.

Supplemental Table 2. Immunohistochemical receptor constellation.

Expression	Estrogen receptor (ER)	Progesterone receptor (PR)	HER 2
**Negative, n (%)**	26 (16)	47 (29)	128 (88)
**Low-positive, n (%)**	3 (2)	11 (7)	Not applicable
**Positive, n (%)**	131 (82)	102 (64)	18 (12)

Receptor expression is considered negative when the level of expression is 1% and lower, low-positive from 1 to 9% and, concerning estrogen and progesterone receptor expression, positive when the level is 10% and higher and in HER2 expression, when exceeding 1%. HER2 is not assessed in precancerous lesions, which have been excluded from the evaluation of HER2 category. HER2: Human Epidermal Growth Factor 2.
